# Acute Mesenteric Ischemia Mimicking Severe COVID-19 Pneumonia: A Case Report on Diagnostic Challenges and Management

**DOI:** 10.7759/cureus.60466

**Published:** 2024-05-16

**Authors:** Tsubasa Nakai, Daisuke Son

**Affiliations:** 1 Department of Community-Based Family Medicine, Faculty of Medicine, Tottori University, Yonago, JPN; 2 Department of General Medicine, Hino Hospital Association Hino Hospital, Hino-gun, JPN

**Keywords:** systemic symptoms, differential diagnosis, abdominal imaging, acute mesenteric ischemia, covid-19

## Abstract

This article demonstrates the need for abdominal imaging in COVID-19 patients with systemic symptoms in the differential diagnosis of acute mesenteric ischemia and critical COVID-19 pneumonia. We detail the case of a 91-year-old man, initially diagnosed with severe COVID-19 pneumonia, who was later found to have acute mesenteric ischemia through abdominal CT imaging, despite lacking typical abdominal symptoms. Abdominal CT revealed intramural and portal emphysema, leading to a diagnosis of acute mesenteric ischemia. Given the patient's advanced age and poor condition, supportive care was chosen, with the patient passing away 12 hours post-admission. This case highlights the critical need for comprehensive evaluation, including abdominal imaging, in COVID-19 patients with systemic symptoms to identify other serious conditions like acute mesenteric ischemia, especially in the absence of specific abdominal pain. Early detection is vital for appropriate management and improved patient outcomes.

## Introduction

Coronavirus disease 2019 (COVID-19) causes a wide variety of systemic symptoms. The most common symptoms are respiratory, causing viral upper respiratory tract infection and viral pneumonia. However, it also causes various systemic complications, including acute respiratory distress syndrome, arrhythmias, cardiac injury, shock, and acute stroke [1]. In particularly severe cases of COVID-19 infection, symptoms may include thromboembolic complications [2]. Acute mesenteric ischemia associated with COVID-19 is a serious complication with an average mortality rate as high as 50% [3]. It requires urgent diagnosis and treatment. However, acute mesenteric ischemia is a rare complication of COVID-19, occurring in only about 3% of hospitalized severe cases [4]. Symptoms of acute mesenteric ischemia include abdominal pain without abdominal tenderness, nausea, vomiting, fever, and anorexia. Blood tests show elevated leukocytes, C-reactive protein (CRP), D-dimer, and lactate. However, all symptoms and blood tests are nonspecific, and early diagnosis is not easy [5]. In this report, we describe a case of acute mesenteric ischemia that was initially diagnosed as severe COVID-19 pneumonia.

## Case presentation

A 91-year-old man with dementia was rushed to the hospital due to fever for the past 24 hours, poor food intake, difficulty moving his body which started on the day of presentation, and mild disturbance of consciousness. He was not unresponsive but showed a noticeable worsening from his usual level of cognitive function due to dementia, characterized by delayed responses and inability to maintain attention. He has a history of atrial fibrillation and has been on long-term medication with edoxabantosilate hydrate (15 mg) and bisoprolol fumarate (2.5 mg) for over 10 years. He remains independent in his activities of daily living. His vital signs at presentation were as follows: temperature 38.5℃, blood pressure 88/70 mmHg, heart rate (HR) 180 bpm, and respiratory rate 40 breaths per minute. Oxygen saturation was 95% with 5 L/min oxygen administration. Despite the administration of 5 L/min of oxygen via a nasal cannula, the arterial blood gas analysis showed a partial pressure of oxygen (pO_2_) of 75 mmHg, which is at the lower boundary of the normal range, indicating that the patient's oxygenation status was precarious. Neurological assessment using the Glasgow Coma Scale scored E4V4M5, totaling 13. Pupillary light reflexes were normal with no asymmetry noted between the left and right pupils. Physical examination revealed breath sounds were vesicular in both pulmonary hemispheres, and there was no lower leg edema. No abdominal tenderness was noted. Clinical examination revealed arterial blood gas with metabolic acidosis with hypoxemia and increased anion gap. Blood tests showed elevated white blood cell count and multiple organ failure (Table 1).

**Table 1:** Laboratory data MCV: mean corpuscular volume, AST: aspartate aminotransferase, ALT: alanine aminotransferase, ALP: alkaline phosphatase, γ-GTP: γ-glutamyl transpeptidase, BUN: blood urea nitrogen, CK: creatine kinase, CRP: C-reactive protein, PT-INR: prothrombin time-international normalized ratio, APTT: activated partial thromboplastin time

Parameter	Result	Reference value
Peripheral blood		
White blood cells	19000	3300-8600 (/μL)
Red blood cells	589	435-555 (×10^4/μL)
Hemoglobin	16.8	13.7−16.8 (g/dL)
Hematocrit	53.5	40.7−50.1(％)
MCV	90.5	83.6-98.2 (fL)
Platelets	29.3	15.8-34.8 (×10^4/μL)
Neutrophils	84	37-72 (%)
Eosinophils	0	0-6 (%)
Basophils	0.1	0-1 (%)
Monocytes	7.6	0-14 (%)
Total protein	7.9	6.6-8.1 (g/dL)
Albumin	3.6	4.1-5.1 (g/dL)
AST	293	13-30 (IU/L)
ALT	95	10-42 (IU/L)
ALP	81	39-113 (U/L)
γ-GTP	18	13-64 (IU/L)
Total bilirubin	1.3	0.4-1.5 (mg/dL)
BUN	109	8-20 (mg/dL)
Creatinine	4.59	0.65-1.07 (mg/dL)
CK	6521	59-248 (IU/L)
Na	156	138-145 (mEq/L)
K	4.5	3.6-4.8 (mEq/L)
Cl	119	101-108 (mEq/L)
Glucose	194	73-109 (mg/dL)
CRP	0.32	0-0.14 (mg/dL)
PT-INR	1.95	0.9-1.1 (-)
APTT	28.9	24.6-33.5 (sec)
Arterial blood gas		
pH	7.371	7.35-3.45 (-)
pO_2_	145	75-100 (mmHg)
pCO_2_	23.6	35-45 (mmHg)
HCO_3_-	13.4	20-26 (mmol/L)
Anion Gap	37.8	10-18 (mmol/L)

EKG showed HR 202/m, atrial fibrillation, and polymerase chain reaction (PCR) test was positive for severe acute respiratory syndrome coronavirus 2 (SARS-CoV-2). Based on respiratory failure, shock, and multiple organ failure, the patient was diagnosed with severe COVID-19 pneumonia without waiting for imaging studies, and early intervention was decided.

**Figure 1:**
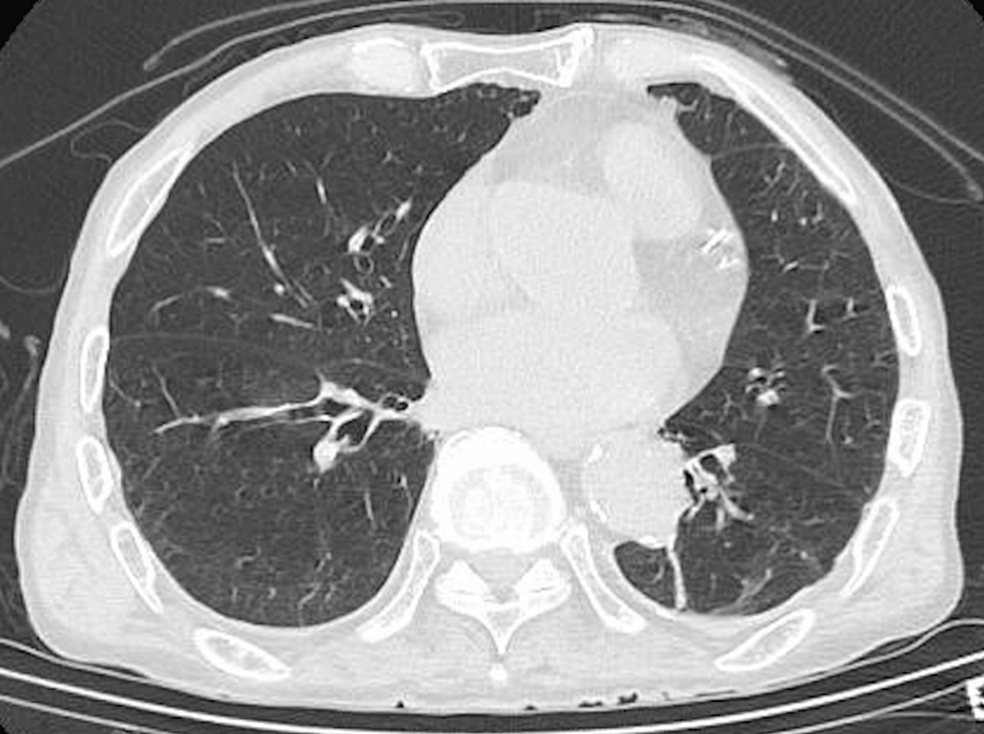
Chest CT which shows no infiltrative shadows

**Figure 2:**
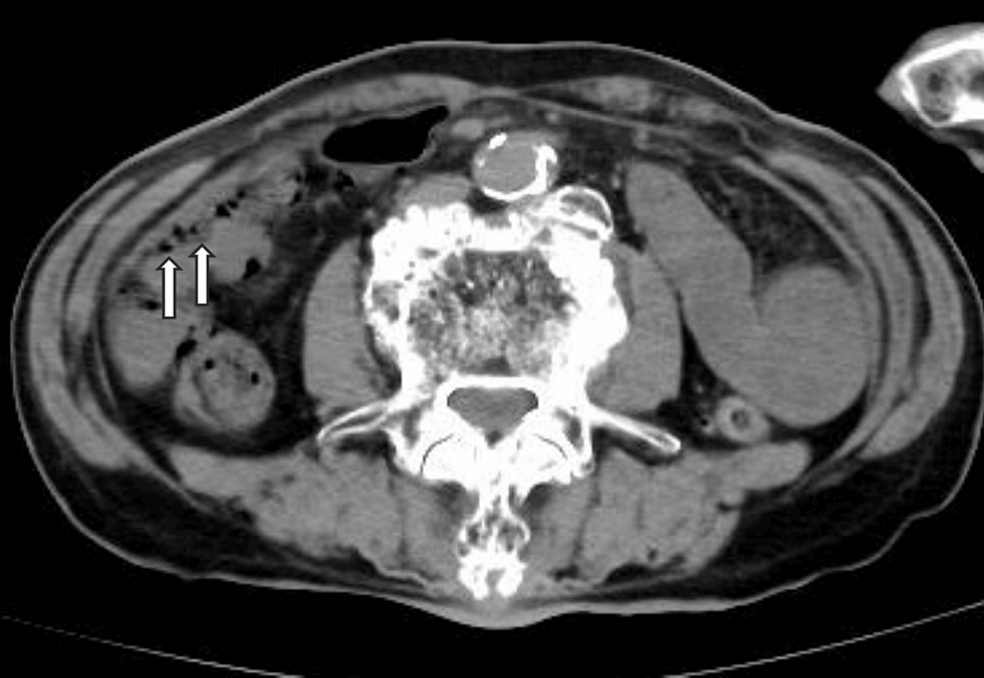
Intramural emphysema of the ileum shown on abdominal CT

**Figure 3:**
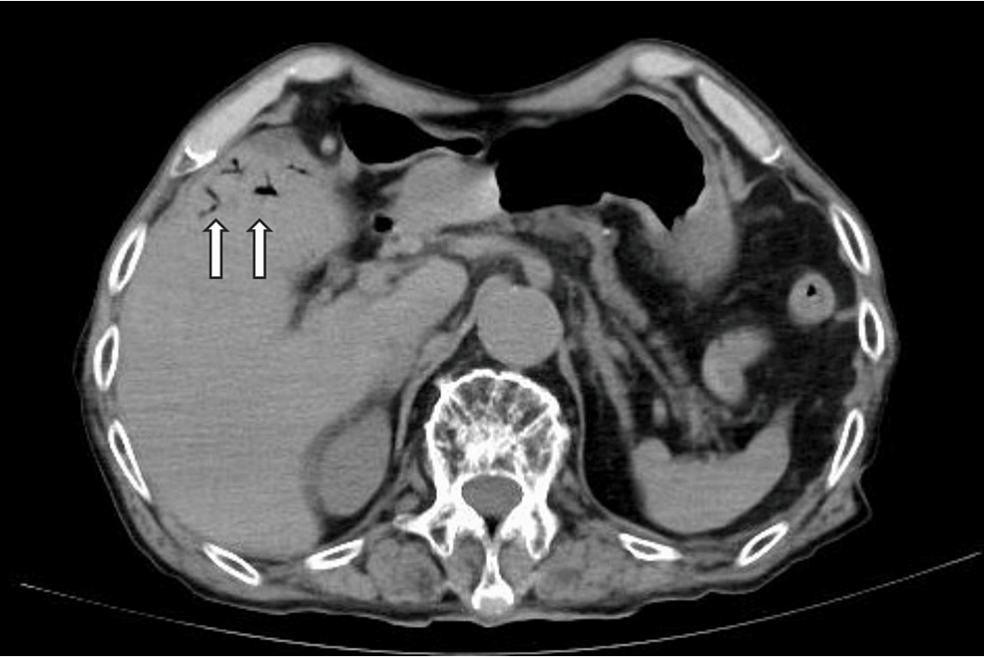
Portal emphysema in the portal vein shown on abdominal CT

**Figure 4:**
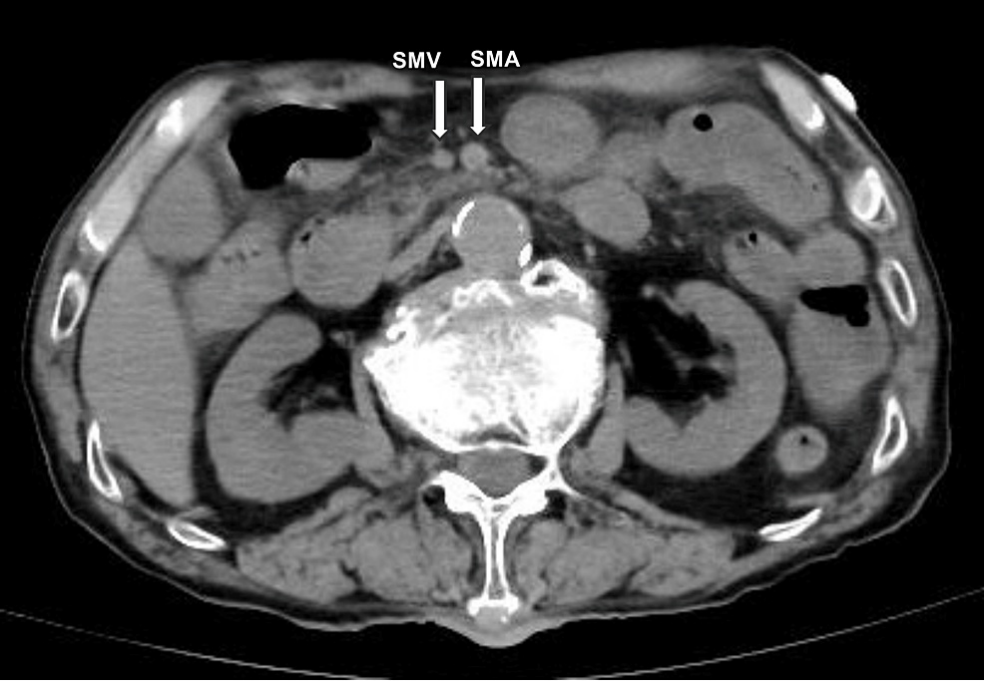
Smaller SMV sign shown on abdominal CT SMA: superior mesenteric artery, SMV: superior mesenteric vein

The patient was admitted to a negative pressure room with airborne infection control. Considering the potential for bacterial pneumonia, blood cultures were obtained, and treatment with a broad-spectrum antibiotic, meropenem 1g every 12 hours, was initiated to address both the suspected pneumonia and to provide prophylactic coverage for other potential bacterial infections given the patient's critical condition. The patient was started on treatment with lemdecivir 200mg per day and anticoagulation with low molecular weight heparin 75 IU/kg per day. His blood pressure stabilized, but he continued to have poor oxygenation. A simple CT scan of the chest and abdomen was performed six hours after admission. No infiltrative shadows were found in the lung fields (Figure 1). An abdominal CT scan was taken to evaluate other causes of the fever, and it showed intramural emphysema in the ileum and portal emphysema in the portal vein from the mesenteric vein (Figures 2, 3), and smaller superior mesenteric vein (SMV) sign (Figure 4).

Based on imaging studies, we diagnosed acute mesenteric ischemia due to COVID-19 rather than severe COVID-19 pneumonia. After consultation with the general surgeon, it was determined that surgical treatment was not indicated due to the patient's overall fragile condition and poor likelihood of tolerating surgery. After discussion with the family, the patient was placed on comfort care measures. The patient was treated with analgesics and sedatives and died 12 hours after admission.

## Discussion

We have experienced a case of COVID-19 infection in an elderly patient initially diagnosed as severe pneumonia but ultimately diagnosed as acute mesenteric ischemia [1,6-12]. Acute mesenteric ischemia is a rare complication of COVID-19 in less than 1% of cases, and there have been a limited number of cases. COVID-19 infection leading to acute mesenteric ischemia is presumed to be a COVID-19-associated hypercoagulable state, von Willebrand factor activation, and direct intestinal endothelial damage due to binding of SAR-CoV-2 to angiotensin-converting enzyme 2 in the intestinal cell epithelium. Hemodynamic instability associated with COVID-19 may also cause non-occlusive intestinal ischemia (NOMI) [13]. The mortality rate of acute mesenteric ischemia is very high. It averages 50% and has been reported to be 100% in patients over 65 years of age [3]. The standard treatment is excision of the necrotic area by laparotomy. Although the diagnosis is likely to be delayed, early diagnosis and early surgical consultation have been reported to improve prognosis [14]. Early diagnosis of acute mesenteric ischemia, however, is often difficult. In most cases, patients present with abdominal pain [5]. However, in severe cases, it may not be possible to make a diagnosis due to the decreased level of consciousness associated with shock, and in the present case, no abdominal pain was observed. In addition, the diagnosis of acute abdomen is further delayed in elderly patients or those with dementia, as was the case in the present patient. This is due to the inability to explain symptoms and to confirm them [15]. In summary, it can be difficult to differentiate acute mesenteric ischemia from COVID-19 pneumonia based on physical and laboratory findings alone in patients with poor complaints of abdominal pain, as in the present case.

When differentiation is difficult, CT imaging of the abdomen can provide clues to distinguish COVID-19 pneumonia from acute mesenteric ischemia. Simple abdominal CT shows thick walls, edema, and dilated bowel (>3 cm). The presence of intestinal emphysema and portal gas suggests intestinal ischemia [3]. If thrombosis of the superior mesenteric artery (SMA) is suspected, contrast-enhanced CT or angiography can be performed to demonstrate stenosis or obstruction of the SMA origin [16]. In the present case, abdominal CT imaging was able to diagnose acute mesenteric ischemia. In severe cases, not only evaluating pneumonia with chest CT but also adding abdominal CT may be helpful in diagnosing, because acute mesenteric ischemia can be an asymptomatic complication of COVID-19.

In conclusion, this case highlights the necessity of considering acute mesenteric ischemia in the differential diagnosis of COVID-19 patients with systemic symptoms but no specific abdominal pain. The strengths of our approach include the prompt use of comprehensive imaging that led to an accurate diagnosis despite the absence of specific symptoms, which is crucial for such atypical presentations. However, the study is limited by its single-case nature, which may not fully represent the broader population. Additionally, while the diagnostic process was effective, it reflects a high-resource setting that might not be available universally, limiting the generalizability of our findings.

## Conclusions

We have experienced a case of acute mesenteric ischemia in a patient initially diagnosed with severe COVID-19 pneumonia. Due to a variety of factors, critical illness COVID-19 pneumonia and acute mesenteric ischemia can be difficult to distinguish on clinical, blood, and physical examination. The presence of bowel wall thickening, edema, bowel dilatation, intestinal emphysema, portal gas, and smaller SMV sign on plain abdominal CT suggests acute mesenteric ischemia. Acute mesenteric ischemia is a rare complication of COVID-19, but physicians should be aware of this condition because a delayed diagnosis can be fatal. When a patient is diagnosed with a severe COVID-19 infection, a head-to-toe physical examination and arterial blood gas as well as an abdominal CT scan are useful for early diagnosis, given the possibility of asymptomatic acute mesenteric ischemia.
